# Supplementary material for: Asymmetric quantum decision-making
Source: Sci Rep. 2023 Sep 5;13:14636. doi: 10.1038/s41598-023-41715-z (PMC10480193; doi:10.1038/s41598-023-41715-z)
Supplement: Supplementary file 1 — Supplementary Information. [file 41598_2023_41715_MOESM1_ESM.pdf]

Supplementary information of asymmetric quantum decision-making

Honoka Shiratori[1,*], Hiroaki Shinkawa[1], André Röhm[1], Nicolas Chauvet[1], Guillaume Bachelier[2], Jonathan Laurent[2], Ryoichi Horisaki[1], and Makoto Naruse[1]

[1]The University of Tokyo, Department of Information Physics and Computing, Graduate School of Information Science and Technology, Tokyo, 113-8656, Japan
[2]Université Grenoble Alpes, CNRS, Institut Néel, Grenoble, 38042, France
[*]8308123768hs@g.ecc.u-tokyo.ac.jp

ABSTRACT

In this supplementary information, we summarize the details of the theoretical elements discussed in the main text.

1 Boundary of the feasible zone (Figure 3)

1.1 Asymmetric OAM system (Figure 3a)

The boundary of the feasible pairs of p_{12} and p_{21} in the case of the asymmetric OAM system obtained using Eq. (19) in the main text was derived. First, the upper bound of $p_{12}+p_{21}$ was proved to be $(1+(p_{12}-p_{21})^2)/2$. In particular,

$$p_{12}+p_{21} \leq \frac{1+(p_{12}-p_{21})^2}{2}. \tag{1}$$

Based on the probabilities listed in Table 1 in the main text,

$$\begin{aligned}\frac{1+(p_{12}-p_{21})^2}{2} &- (p_{12}+p_{21}) \\ &= \frac{1}{2}(a_2b_1-a_1b_2-1)(a_2b_1-a_1b_2+1)\{2\alpha\beta(a_2b_1+a_1b_2)-1\}\{2\alpha\beta(a_2b_1+a_1b_2)+1\}.\end{aligned} \tag{2}$$

Herein, because a_1 and a_2 satisfy $a_1^2+a_2^2=1$, θ_a can be introduced such that $a_1=\cos\theta_a,\ a_2=\sin\theta_a$. Similarly, θ_b can be introduced such that $b_1=\cos\theta_b,\ b_2=\sin\theta_b$. Meanwhile, with θ, let $\alpha=\sin\theta$ and $\beta=\cos\theta$. Therefore,

$$a_2b_1-a_1b_2=\sin\theta_a\cos\theta_b-\cos\theta_a\sin\theta_b=\sin(\theta_a-\theta_b), \tag{3}$$

$$a_2b_1+a_1b_2=\sin\theta_a\cos\theta_b+\cos\theta_a\sin\theta_b=\sin(\theta_a+\theta_b), \tag{4}$$

$$\alpha\beta=\sin\theta\cos\theta=\frac{1}{2}\sin 2\theta. \tag{5}$$

Applying Eqs. (3) to (5) into Eq. (2), we obtain:

$$\frac{1+(p_{12}-p_{21})^2}{2}-(p_{12}+p_{21})=\frac{1}{2}\{\sin^2(\theta_a-\theta_b)-1\}\{\sin^2 2\theta\sin^2(\theta_a+\theta_b)-1\}. \tag{6}$$

Because $0\leq\sin^2(\theta_a-\theta_b)\leq 1$, $0\leq\sin^2 2\theta\leq 1$, $0\leq\sin^2(\theta_a-\theta_b)\leq 1$,

$$\sin^2(\theta_a-\theta_b)-1\leq 0,\ \ \sin^2 2\theta\sin^2(\theta_a-\theta_b)-1\leq 0 \tag{7}$$

holds. Therefore the following is true,

$$0\leq\frac{1+(p_{12}-p_{21})^2}{2}-(p_{12}+p_{21}) \tag{8}$$

Thus, Eq. (1) is proven. As $\frac{1+(p_{12}-p_{21})^2}{2} - (p_{12}+p_{21})$ cannot be negative, $p_{12}+p_{21}$ obviously cannot be larger than $\frac{1+(p_{12}-p_{21})^2}{2}$. Therefore, the boundary in Figure 3a is proven to be $\frac{1+(p_{12}-p_{21})^2}{2}$. Equation (1) holds when $\theta_a, \ \theta_b, \ \theta$ is represented by integers $n, \ m, \ k$, as follows.

$$\theta_a = \frac{(n+m+1)\pi}{2}, \ \ \theta_b = \frac{m-n}{2}\pi, \ \ \theta = \frac{2k+1}{4}. \tag{9}$$

Therefore, the upper bound of $p_{12}+p_{21}$ is proven to be $(1+(p_{12}-p_{21})^2)/2$. In addition, the lower bound of $p_{12}+p_{21}$ is 0. As $p_{12}+p_{21}$ is continuous, $p_{12}+p_{21}$ takes all values from 0 to $(1+(p_{12}-p_{21})^2)/2$:

$$0 \leq p_{12}+p_{21} \leq \frac{1+(p_{12}-p_{21})^2}{2}. \tag{10}$$

By considering the condition of p_{12} and p_{21}, , evidently:

$$p_{12} \geq 0, \ \ p_{21} \geq 0, \tag{11}$$

because p_{12} and p_{21} are probabilities. Based on the condition of Eq. (10) and (11), the feasible pairs of p_{12} and p_{21} are proven to be the blue-colored areas in Figure 3a in the main text. ■

1.2 Asymmetric entangled photon decision maker (Figure 3b)

As proven in the main text that

$$p_{12} = \frac{\sin^2\beta_Y \cos^2\alpha_X}{2}, \ \ p_{21} = \frac{\sin^2\beta_X \cos^2\alpha_Y}{2}. \tag{12}$$

Because α_X, α_Y, β_X, and β_Y are independent,

$$0 \leq p_{12} \leq \frac{1}{2}, \ \ 0 \leq p_{21} \leq \frac{1}{2}. \tag{13}$$

Provided that Eq. (13) holds, p_{12} and p_{21} can take any value owing to the continuity of trigonometric functions. Therefore, the boundary by the asymmetric entangled photon decision maker in Figure 3b is proven. ■

1.3 OAM attenuation (Figure 3c)

As explained in the main text that

$$p_{12} = \left(a_1^2b_2^2 + a_2^2b_1^2 - 2a_1a_2b_1b_2\cos(\theta_1-\theta_2)\right) d_{aX}^2 d_{bY}^2, \ \ p_{21} = \left(a_2^2b_1^2 + a_1^2b_2^2 - 2a_1a_2b_1b_2\cos(\theta_1-\theta_2)\right) d_{aY}^2 d_{bX}^2. \tag{14}$$

They have $a_1^2b_2^2 + a_2^2b_1^2 - 2a_1a_2b_1b_2\cos(\theta_1-\theta_2)$ in common; however, the attenuation rates multiplied are different. As the following is true:

$$0 \leq a_1^2b_2^2 + a_2^2b_1^2 - 2a_1a_2b_1b_2\cos(\theta_1-\theta_2) \leq \frac{1}{4}, \tag{15}$$

and owing to the fact that d_{aX}, d_{aY}, d_{bX}, and d_{bY} are independent, the boundary by the asymmetric entangled photon decision maker in Figure 3c is proven. ■

2 Boundary of asymmetric ratio and loss plus conflict probability (Figure 4)

2.1 Asymmetric OAM system (Figure 4a)

In case of Figure 3a (in the main text), points on the boundary have the maximum $p_{12}+p_{21}$, provided p_{21}/p_{12} is consistent. The values of loss probability plus conflict probability are minimum on the boundary for the same p_{21}/p_{12}. Therefore, if the boundary in Figure 3a can be converted to that in Figure 4a (in the main text), then the boundary in Figure 4a is mathematically derived. This conversion is shown as follows.

Let y be the ratio of p_{12} and p_{21}: $p_{21} = yp_{12}$. Let x be loss probability plus conflict probability on the boundary in Figure 3b. Here, x is $p_{12}+p_{21}$ on the intersection of the boundary in Figure 3a and line of $p_{21} = yp_{12}$. We refer to this intersection as V_1. On V_1,

$$2(1+y)p_{12} = 1+(1-y)^2p_{12}^2, \tag{16}$$

$$(1-y)^2 p_{12}^2 - 2(1+y)p_{12} + 1 = 0, \tag{17}$$

$$p_{12} = \frac{1+y \pm 2\sqrt{y}}{(1-y)^2} = \frac{(1 \pm \sqrt{y})^2}{(1-y)^2}. \tag{18}$$

If

$$p_{12} = \frac{(1+\sqrt{y})^2}{(1-y)^2}, \tag{19}$$

then,

$$x = 1-(p_{12}+p_{21}) = 1 - \frac{y+1}{(\sqrt{y}-1)^2} = \frac{-2\sqrt{y}}{(\sqrt{y}-1)^2} < 0. \tag{20}$$

This is strange because x should satisfy $0 \leq x \leq 1$. Therefore, the sign in Eq. (18) is determined:

$$p_{12} = \frac{(1-\sqrt{y})^2}{(1-y)^2}. \tag{21}$$

Hence,

$$x = 1-(p_{12}+p_{21}) = 1 - \frac{y+1}{(\sqrt{y}+1)^2} = \frac{2\sqrt{y}}{(\sqrt{y}+1)^2}. \tag{22}$$

By solving Eq. (22), we obtain:

$$y = \begin{cases} \dfrac{(1+\sqrt{1-2x})^2}{(1-\sqrt{1-2x})^2} & \text{when } y \geq 1, \\ \\ \dfrac{(1-\sqrt{1-2x})^2}{(1+\sqrt{1-2x})^2} & \text{when } y \leq 1. \end{cases} \tag{23}$$

Thus, Eq. (20) in the main text is derived. ■

2.2 Asymmetric entangled photon decision maker (Figure 4b)

In Figure 3b, points on the boundary have the maximum $p_{12} + p_{21}$ and minimum loss probability plus conflict probability for the same p_{21}/p_{12}, as in the previous subsection. If the boundary in Figure 3b can be converted to the boundary in Figure 4b, then that in Figure 4a is mathematically derived. This conversion is shown as follows.

Let y be the ratio of p_{12} and p_{21}: $p_{21} = yp_{12}$. Let x be loss probability plus conflict probability on the boundary in Figure 3b. First, consider the case where y is equal to or greater than 1. In this case,

$$p_{12} = \frac{1}{2y}, \quad p_{21} = \frac{1}{2}. \tag{24}$$

Therefore,

$$x = 1-(p_{12}+p_{21}) = 1 - \left(\frac{1}{2y} + \frac{1}{2}\right) \tag{25}$$

$$= \frac{y-1}{2y}. \tag{26}$$

By solving Eq. (26) about y, we obtain:

$$y = \frac{1}{1-2x}. \tag{27}$$

Next, when y is equal to or smaller than 1,

$$p_{12} = \frac{1}{2}, \ \ p_{21} = \frac{y}{2}. \tag{28}$$

Therefore,

$$x = 1 - (p_{12} + p_{21}) = 1 - \left(\frac{1}{2} + \frac{y}{2}\right) \tag{29}$$

$$= \frac{1-y}{2}. \tag{30}$$

By solving Eq. (30) about y, we obtain:

$$y = 1 - 2x. \tag{31}$$

Therefore, the mathematical formula of the boundary is expressed as:

$$y = \begin{cases} \dfrac{1}{1-2x} & \text{when } y \geq 1, \\ 1-2x & \text{when } y \leq 1. \end{cases} \tag{32}$$

This corresponds to Eq. (35) in the main text. ■

2.3 OAM attenuation (Figure 4c)

Next, the conversion from the boundary in Figure 3c to that in Figure 4c is as follows. Let y be the ratio of p_{12} and p_{21}: $p_{21} = yp_{12}$. Let x be loss probability plus conflict probability on the boundary in Figure 3b. First, consider the case where y is equal to or greater than 1. In this case,

$$p_{12} = \frac{1}{4y}, \ \ p_{21} = \frac{1}{4}. \tag{33}$$

Therefore,

$$x = 1 - (p_{12} + p_{21}) = 1 - \left(\frac{1}{4y} + \frac{1}{4}\right) \tag{34}$$

$$= \frac{3y-1}{4y}. \tag{35}$$

By solving Eq. (35) about y, we obtain:

$$y = \frac{1}{3-4x}. \tag{36}$$

Next, when y is equal to or smaller than 1,

$$p_{12} = \frac{1}{4}, \ \ p_{21} = \frac{y}{4}. \tag{37}$$

Therefore,

$$x = 1 - (p_{12} + p_{21}) = 1 - \left(\frac{1}{4} + \frac{y}{4}\right) \tag{38}$$

$$= \frac{3-y}{4}. \tag{39}$$

By solving Eq. (39) about y, we obtain:

$$y = 3 - 4x. \tag{40}$$

Therefore, the mathematical formula of the boundary is expressed as:

$$y = \begin{cases} \dfrac{1}{3-4x} & \text{when } y \geq 1, \\ 3-4x & \text{when } y \leq 1, \end{cases} \tag{41}$$

Thus, Eq. (39) in the main text is derived. ■
